# Inactivated vaccine Covaxin/BBV152: A systematic review

**DOI:** 10.3389/fimmu.2022.863162

**Published:** 2022-08-09

**Authors:** Tousief Irshad Ahmed, Saqib Rishi, Summaiya Irshad, Jyoti Aggarwal, Karan Happa, Sheikh Mansoor

**Affiliations:** ^1^ Department of Clinical Biochemistry, Sher-I-Kashmir Institute of Medical Sciences, Srinagar, JK, India; ^2^ Department of Microbiology, Government Medical College, Srinagar, JK, India; ^3^ Department of Ophthalmology, Government Medical College, Jammu, JK, India; ^4^ Department of Biochemistry, Maharishi Markandeshwar Institute of Medical Sciences and Research (MMIMSR), Ambala, HR, India; ^5^ Department of General Medicine, Sher-I-Kashmir Institute of Medical Sciences, Srinagar, JK, India; ^6^ Advanced Centre for Human Genetics, Sher-I-Kashmir Institute of Medical Sciences, Srinagar, JK, India

**Keywords:** BBV152, Covaxin, inactivated vaccine, Covishield, efficacy, immunogenicity, safety

## Abstract

We systematically reviewed and summarized studies focusing on Bharat Biotech’s Whole Virion Inactivated Corona Virus Antigen BBV152 (Covaxin), which is India’s indigenous response to fighting the SARS-CoV-2 pandemic. Studies were searched for data on the efficacy, immunogenicity, and safety profile of BBV152. All relevant studies published up to March 22, 2022, were screened from major databases, and 25 studies were eventually inducted into the systematic review. The studies focused on the virus antigen (6 μg) adjuvanted with aluminium hydroxide gel and/or Imidazo quinolin gallamide (IMDG), aTLR7/8 agonist. Pre-clinical, phase I, and II clinical trials showed appreciable immunogenicity. Both neutralizing and binding antibody titers were significant and T cell responses were Th1-biased. Phase III trials on the 6 μg +Algel-IMDG formulation showed a 93.4% efficacy against severe COVID-19. Data from the trials revealed an acceptable safety profile with mostly mild-moderate local and systemic adverse events. No serious adverse events or fatalities were seen, and most studies reported milder and lesser adverse events with Covaxin when compared with other vaccines, especially Oxford-Astra Zeneca’s AZD1222 (Covishield). The immunogenicity performance of Covaxin, which provided significant protection only after the second dose, was mediocre and it was consistently surpassed by Covishield. One study reported adjusted effectiveness against symptomatic infection to be just 50% at 2 weeks after the second dose. Nonetheless, appreciable results were seen in previously infected individuals administered both doses. There was some evidence of coverage against the Alpha, Beta, and Delta variants. However, neither Covaxin nor Covishield showed sufficient protection against the Omicron variant. Two studies reported super-additive results on mixing Covaxin with Covishield. Further exploration of heterologous prime-boost vaccination with a combination of an inactivated vaccine and an adenoviral vector-based vaccine for tackling future variants may be beneficial.

## Introduction

With SARS-CoV-2 infections affecting more than half a billion and resulting in around 6.3 million deaths at the time of writing, more than 220 countries faced a monumental task in combating the devastation inflicted by this virus (1). Extensive vaccination efforts were initiated and successfully concluded all over the world in record times. Unfortunately, the huge number of cases of SARS-CoV-2 worldwide provided fertile ground for genomic changes precipitating the emergence of newer variants, such as the Alpha variant (B.1.1.7), which emerged in the UK, Beta variant (B.1.351) in South Africa, Gamma (P.1) in Brazil, Epsilon (B.1.429) emerging from California, and Iota (B.1.526) in New York. The phenomenal increase in SARS-CoV-2 infections in the Indian subcontinent during the second wave and the flouting of social distancing norms at religious and political gatherings provided the opportunity for the emergence of new variants, the Delta and Kappa (1.617.1 and 1.617.2, respectively) emerging in the country around December 2020 (2).

Bharat Biotech’s Covaxin (BBV152) was the second-most administered vaccine in India at 327 million doses received at the time of writing (3). It is a β-propiolactone inactivated vaccine (4). The inactivated whole-virion structure is combined with an adjuvant, Imidazo -quinoline gallamide, which is a toll-like receptor 7/8 agonist molecule adsorbed to alum (Algel-IMDG). The formulation improves homing of vaccine antigen onto draining lymph nodes without systemic spillage. A genetically stable strain NIV-2020-770 containing the Asp614Gly mutation used for making Covaxin was isolated from an asymptomatic SARS-CoV-2 positive patient at the National Institute of Virology (NIV), Pune (5). Biosafety level 3 (BSL3) manufacturing facilities with a vero cell manufacturing platform were utilized in the manufacturing process (6). The strain used is 99.7% identical to Wuhan Hu-1 (7). Five to 10% newborn calf serum in Dulbecco’s Modified Eagle medium (DMEM) was used to grow Vero CCL-81 cells in tissue culture flasks and cell stacks. Further virus propagation was achieved in bioreactors which maintained a temperature of 36 ± 1°C. Harvesting was done at 36-72 h post-infection and supernatants were processed. Additional purification and concentration were done by column chromatography and a tangential flow filtration system, respectively (8). The purified final bulk obtained from the inactivation procedure has been found to contain spike and nucleocapsid protein. Transmission electron microscopy (TEM) shows intact, oval structures with the characteristic crown shape (7). Covaxin got early approval from Indian Drug regulatory agencies (9). Studies on vaccine safety have always presented challenges. Antibody-dependent enhancement (ADE) has been a worrying concern with inactivated vaccines and the changes in conformations of spike proteins on inactivation with β-propiolactone may be a cause for concern (10). Autoimmune glomerular disorders have been reported 2 weeks after vaccination with Covaxin (11). A case of Cutaneous small-vessel vasculitis was reported 5 days after inoculation (12). There was also a report of varicella zoster reactivation in a 72-year-old woman a week after receiving the vaccine (13). Coronary thromboembolic phenomena have also been seen, though on a much lesser scale compared to other vaccines (14). Nonetheless, the WHO accorded Emergency use listing (EUL) approval to Covaxin on 3 November 2021 after several delays, its Strategic Advisory Group of Experts on Immunization (SAGE) having previously recommended two doses spaced 4 weeks apart in all adults (15). Several South American and African nations have also been using it in their programs, though not without reservations (16, 17).

We aim to systematically review the overall efficacy, immunogenicity, and safety of BBV152 Covaxin vaccine, which could potentially guide public health policy in relation to combating the threat posed by SARS-CoV-2, especially in those countries that are actively considering adding it to their regimens.

## Methods

### Search strategy

**Figure 1:**
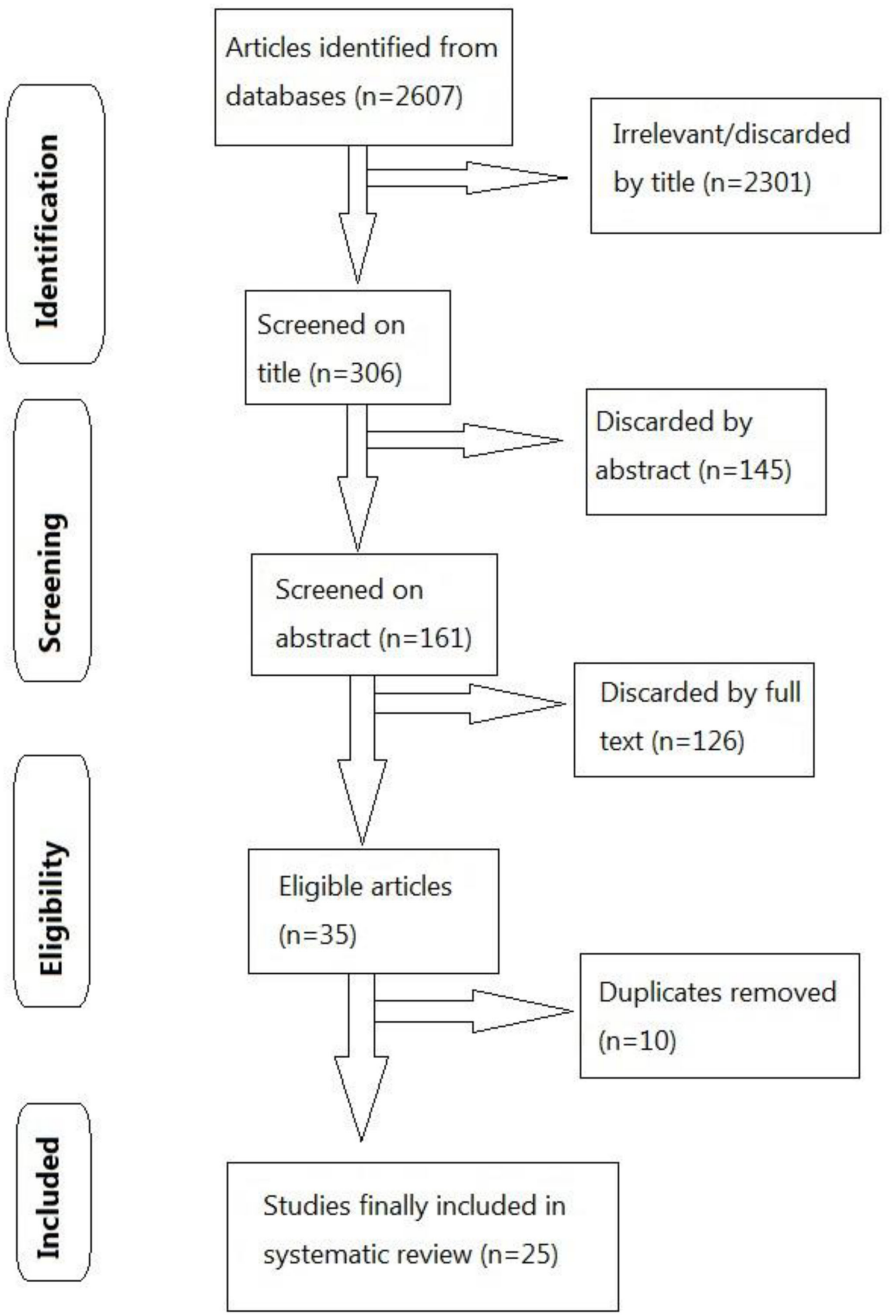
Flow diagram utilizing PRISMA (Preferred Reporting Items for Systematic Reviews and Meta-analyses) for study selection.

The Preferred Reporting Items for Systematic Reviews and Meta-Analyses Statement (PRISMA) recommendations were followed in this analysis. (**Figure 1**) A systematic literature search with no language restriction was performed in electronic databases, including PubMed, Google Scholar, Directory of Open Access Journals (DOAJ), as well as Lancet, to identify eligible studies published up to March 22, 2022. The search strategy was based on the following keywords and MeSH terms: “BBV152”, “Covaxin”, “vaccine”, “vaccination”, “safety” and “efficacy”. Reference lists of selected studies were also screened. In addition, internet engines were utilized to search for web pages that might have references of interest.

### Study selection

Two investigators (TIA and SR) independently performed the literature screening to identify eligible studies. Studies eligible for inclusion were studies of any design (case–control, case–cohort, prospective cohort, randomized control trials, cross-sectional, human, as well as non-human studies), which reported the effectiveness of the BBV152 vaccine to prevent reverse transcription–polymerase chain reaction (RT-PCR) confirmed COVID-19 (through comparison between vaccinated and unvaccinated individuals) and adjusted for covariates. For multiple studies based on the same data, or where preprints were succeeded by publication in indexed journals, the most recent ones were mentioned. Studies involving heterologous administration of BBV152 with other vaccines had interesting results and were included. One study focusing on chemokine and cytokine subsets elicited was not excluded. Studies comparing BBV152 with other vaccines or involving individuals previously infected with SARS-CoV-2 were included.

### Exclusion criteria

We excluded studies that reported unadjusted effectiveness estimates, or which did not use RT-PCR to confirm the diagnosis of COVID-19. Uncorrected manuscripts and pre-prints were not included. One study focusing on breakthrough infections had to be excluded as data for both Covaxin and another vaccine codenamed AZD1222 (brand name Covishield) were grouped together and individual data for Covaxin could not be retrieved (18). Two other studies also had to be excluded due to a lack of Covaxin-specific subgroup analysis (19, 20).

Studies were presented chronologically wherever possible.

We had three outcomes of interest, BBV-152

(a) vaccine efficacy, which is defined as ‘a proportionate reduction in disease attack rate (AR) between the unvaccinated (ARU) and vaccinated (ARV) study cohorts’ (21),

(b) immunogenicity of the vaccine, measured by estimating either binding antibodies by Enzyme-linked immunosorbent assays (ELISA) or Neutralizing Antibodies (NAb), by plaque reduction neutralization assays (PRNT_90_ or PRNT_50_), focus reduction neutralization titer (FRNT_50_), or microneutralization assay (MNT_50_), as well as cytokine and chemokine profiles, and

(c) vaccine safety.

Studies examining either efficacy, immunogenicity, or safety, or any combination of the three, were included. Each included study was studied independently by two investigators (TIA and SR), who also obtained details of the same under the headings of first author’s surname, study design, sample population and subgroups, number of participants, the incidence of COVID-19 (either asymptomatic, symptomatic with more than one grade of severity) in both vaccinated and unvaccinated individuals, and adjusted vaccine effectiveness estimates and covariates. Two investigators (SM and SI) utilized the Newcastle-Ottawa Scale to gauge the quality of included observational studies, and a score >7 was considered high quality and suitable for inclusion (22).

## Results

A total of 25 articles met inclusion and quality criteria. Of these, three focused on efficacy, 19 examined immunogenicity, 10 included safety assessments. Three were animal-based studies. Full papers were assessed. Except for two articles based in Iran, all the studies were conducted in India. The articles were published in English between December 2020 and March 22, 2022. Several of the studies had the involvement of employees of Bharat Biotech, the company producing Covaxin.

## Discussion

Inactivated vaccines have been traditionally used successfully for protection against historically notorious diseases like smallpox, polio, rabies, among others. Theoretically, the intact yet inactivated pathogen elicits a broader immune response compared to other platforms. Epitope coverage of inactivated vaccines is extensive and less prone to circumvention by newer variants (8). The pre-clinical animal studies on the Syrian Hamsters and Rhesus macaques suggested satisfactory efficacy and dose-sparing effect of the 3 µg BBV152 vaccine + Algel-IMDG (in comparison to the 6 μg + Algel-IMDG and other combinations). These animal studies showed high Nab titers, displayed prompt viral clearance from the lower respiratory tract, and displayed no evidence of radiographic abnormalities by the 7th day post-inoculation (8, 23). In the third animal study on three species, 100% seroconversion was observed in 21 days and peak titers were seen on day 28. This study showed sufficient levels of binding Ab and Nab lasting up to 98 days after the first dose. The reliability of the Algel-IMDG adjuvant was also established, as it was found to be non-mutagenic and induced Th1-biased antibody response (7).

The phase I trials presented a Th1-skewed response. Post second dose Seroconversion rates of both the Algel-IMDG adjuvanted combinations of 3 μg and 6 μg were found to be comparable (6). Both cell-mediated and humoral immunity appeared to be sufficiently stimulated. An impressive safety profile with local and systemic adverse events in the range of 17%-21% (none severe) paved the way for phase II trials. In both the phase II trials as well as the follow-up to the phase I trials, the 6 µg + Algel-IMDG combination was found to have the highest immunogenicity and was eventually selected for phase III trials (**Table 1**) (24). These trials revealed a 93.4% efficacy against severe COVID-19, although efficacy against the Delta variant was lower at 65.2%. There were minimal lot-to-lot variations and the safety profile was similar to placebo (25). Notwithstanding the impressive safety profile (just 12.4% adverse events) established in the trials, an Iranian study reported 92.9% incidence of adverse events, with most of the complaints being of injection site pain. However, the Iranian study included only 42 Covaxin recipients compared to the much larger number in phase III trials. Also, similarly high adverse events were reported for the other vaccines, Oxford-Astra Zeneca’s AZD1222(Covishield) and Sputnik V, included in the study (10). The COVAT study showed an increased incidence of mild-moderate adverse events after the first dose in Covaxin (31.2%) compared to second dose (11.1%) and a 2.2% breakthrough infection rate. These rates were found to be better than that seen with AZD1222 recipients, who experienced 46.7% adverse events after the first dose, 18.1% after the second dose, and 5.5% breakthrough events (26). Basavaraja et al. also reported lower incidence rates for Covaxin compared to Covishield, mostly related to vaccination anxiety (27). On the other hand, on comparing two equally sized groups, Choudhary et al. reported higher breakthrough events with Covaxin compared to Covishield (28). Sharma et al. also reported a slightly higher breakthrough infection rate with Covaxin compared to Covishield (29). However, in the COVAT follow-up studies, breakthrough infection rates were similar for the two vaccines (30). Incidence rates varied from the high values reported by the Iranian study to the much lower values of 0.57% reported by Basavaraja et al. (27) The latter utilized spontaneous reporting of adverse events and supervised assessment was conducted for only 30 minutes, which might have resulted in under-reporting of any events occurring after that period. In another Iranian study, adenovirus-vector-based recipients, especially those getting Covishield, reported more numerous and intense side effects compared to vaccinees receiving inactivated formulations. This was attributed to the increased elicitation of cytokine/chemokine responses in the viral vector vaccines (31). Suffice to mention that no serious adverse events or deaths were reported with BBV152 in any study we included, and injection site pain was the decisively predominant adverse event.

**Table 1:** Phase I, II & III trials on the BBV152/Covaxin.

Study	Type of study	Number of participants	Study groups involved	Findings on immunogenicity/efficacy	Findings on safety	Additional comments
Ella et al. (6)	Double-blind, randomised, phase 1 trial	100	Covaxin 6 µg with Alum	Post 2^nd^ dose Seroconversion rates (MNT_50_) 82.8% Post 2^nd^ dose Seroconversion rates (PRNT_50_) 86.6%	Local and systemic adverse events in 14 (14%; 8·1–22·7) One report of a serious adverse event	2 dose study (Day 0,14) demonstrated induction of cell-mediated as well as humoral responses. CD4+ and CD8+ T-cell responses detected in 16 from both alum-IMDG groups. Covaxin formulations, especially Alum-IMDG, were Th1 skewed with IgG1/IgG4 ratios>1.
		100	Covaxin 3 µg with Alum-IMDG (Imidazoquinoline class)	Post 2^nd^ dose Seroconversion rates(MNT_50_) 87·9% Post 2^nd^ dose Seroconversion rates (PRNT_50_) 93.4% similar anti-spike, anti- receptor binding, and anti-nucleoprotein IgG titers (GMTs)	Local and systemic adverse events in 17 (17%; 95% CI 10·5–26·1)	
		100	Covaxin 6 µg with Alum-IMDG	Post 2^nd^ dose Seroconversion rates (MNT_50_) 91.9% Post 2^nd^ dose Seroconversion rates (PRNT_50_) 86.4%	Local and systemic adverse events in 21 (21%; 13·8–30·5)	
		75	Algel only (controls)	Post 2^nd^ dose Seroconversion rates(MNT_50_) 8%	Local and systemic adverse events in 10 (10%; 6·9–23·6)	
Ella et al. (24)	double-blind, randomised, multicentre, phase 2 clinical trial	190	3 µg + Algel-IMDG group	Day 56- Geometric mean titers (GMTs; PRNT_50_)- (100·9 [95% CI 74·1–137·4]) Day 56- Geometric mean titers (GMTs; MNT_50_) -(92·5 [95% CI 77·7–110·2]) Seroconversion (based on PRNT_50_) - 92.9% [95% CI 88·2–96·2] Seroconversion (based on MNT_50_) -88% [95% CI 82.4–92.3]	Solicited local and systemic side-effects in 20·0% (95% CI 14·7–26·5) on days 0-7 and 28-35. No serious adverse events	Utilising previous study(6) the 3 μg + Algel-IMDG & 6μg + Algel-IMDG were selected for phase 2 trial. 2 doses given IM(Day 0,28) The 6 µg + Algel-IMDG group had significantly higher GMTs (p=0·0041). Seroconversion defined as ‘a post-vaccination titer at least four times higher than that preceding vaccination.’ T-cell responses were Th1-biased.
		190	6 µg + Algel-IMDG group	Day 56- Geometric mean titers (GMTs; PRNT_50_) (197·0 [95% CI 155·6–249·4]) Day 56- Geometric mean titers (GMTs; MNT_50_)-(160.1 [95% CI 135.8–188.8]) Seroconversion (based on PRNT_50_) - (98·3% [95% CI 95·1–99·6]) Seroconversion (based on MNT_50_) - 96·6% [95% CI 92·8–98·8])	Solicited local and systemic side-effects in 21·1% (95% CI 15·5–27·5) on day 0-7 and 28-35. No serious adverse events	
	Previous phase I trial (6) was subject to **follow-up** at 3 months after 2^nd^ dose (Day 104)		3 µg + Algel-IMDG group	Day 104 GMTs (MNT_50_) - 39·9 (95% CI 32·0–49·9) Seroconversion (based on MNT_50_) – 73.5% [95% CI 63.6–81.9]	No new serious (or otherwise) adverse events between days 42-104 from	NAb titers persisted in all participants at day 104 comparable to convalescent sera. T-cell memory response more vivid in 6 µg + Algel-IMDG group. No significant differences in GMTs between days 42 and 104 across all vaccinated groups
			6 µg + Algel-IMDG group	Day 104 GMTs (MNT_50_) - 69·5 (95% CI 53·7–89·9) Seroconversion (based on MNT_50_) – 81·1% [71·4–88·1]		
			6 µg + alum(Algel) group	Day 104 GMTs (MNT_50_) - 53·3 (95% CI 40·1–71·0) Seroconversion (based on MNT_50_) – 73·1% [62·9–81·8]		
			Algel only	Day 104 GMTs (MNT_50_) - 20·7 (95% CI 14·5–29·5)		
Ella et al. (25)	Randomised (phase 3) clinical trial	12221	6 µg + Algel-IMDG group	24 symptomatic COVID-19 cases out of 8471 recipients Efficacy: Any severity COVID-19-77.8% Asymptomatic COVID19- 63·6% Severe COVID-19 (as per FDA definition)- 93·4% Against Delta-variant- 65.2% Day 56 GMTs (MNT_50_) – 125.6 Day 56 GMTs (ELISA U/ml): 9742 for S1 protein, 4124 for RBD, 4161 for N protein	1597 adverse events [12·4%] No anaphylaxis/vaccine-related deaths	2 doses (Day 0,28)of 6 µg + Algel-IMDG given . Primary outcome geared to finding efficacy in preventing symptomatic RTPCR +ve COVID-19. Immune responses were found to be independent of age. Lot to lot variations of vaccine batches found insignificant.
		12198	Placebo	106 symptomatic COVID-19 cases out of 8502 recipients Day 56 GMTs (MNT_50_) – 13.7 Day 56 GMTs (ELISA U/ml): No change from baseline	1597 adverse events [12·4%]	

Immunogenicity assessments of Covaxin did not usually outperform that observed with other vaccines. In the COVAT study, Covishield surpassed BBV152 in the observed NAb titers and seropositivity rates after the first dose itself, which was barely equaled even after two doses of Covaxin had been administered (**Table 2**). Among fully vaccinated recipients, Covaxin could elicit an anti-spike Ab geometric mean titer (GMT) of only 48.3 AU/ml, which was less than half that observed with Covishield. The former also elicited a seropositivity of anti-Spike Ab of just 80% compared to 98.1% observed in the latter (26). This superiority in seroprevalence and peak GMT of Covishield was maintained at all time points from 1 to 6 months after the second dose, as seen in the follow-up to the COVAT study. Nonetheless, the declines in peak values were just as rapid, so that by the end of 6 months post the second dose, there was a narrowing of the Ab titer gap between the two vaccines. While Covishield showed a peak of almost 100% seropositivity 3 weeks after the second dose, Covaxin showed peak seropositivity of less than 80% (30). Dash et al. similarly reported higher seropositivity rates with Covishield, the IgG titers against Spike protein being three times that seen with Covaxin. However, they also reported a breakthrough infection related fatality with Covishield (32). The superiority of Covishield over BBV152 was again demonstrated by Choudhary et al., which showed several-fold higher elicitation of spike protein IgG by the former vaccine over the latter. The study noted a four-fold reduction in spike protein Ab titers at 6 months after the second dose for Covaxin, while Covishield showed only a two-fold reduction, which was at variance with the COVAT follow-up results. However, Choudhary et al. had a much higher number of vaccinees receiving Covaxin compared to the COVAT study, so could be considered more reliable. Both studies, however, agree on the consistently higher titers of Covishield at all time points. Additionally, in previously unexposed seronegative individuals, an 81.9% seroconversion at 4 weeks after the first dose was observed with Covishield compared to just 16.1% with Covaxin (28). This was also proven in the study by Malhotra et al., which reported poor immunogenicity in individuals partially immunized with BBV152. Unlike Covishield, where antibody titers started peaking quickly after the first dose itself, it required at least two doses for Covaxin to be anywhere near as effective. In participants with prior viral exposure, two doses of BBV152 accorded sufficient protection, with an 87% efficiency against symptomatic reinfection, while a single dose was only 16% effective (43). A questionnaire-based study of health-care workers reported significantly reduced incidence and severity of COVID-19 infection in those receiving two doses of both Covaxin and Covishield compared to a single dose of either vaccine (44). Kumar et al. demonstrated the induction of innate, adaptive immune responses, as well as cytokine and chemokine induction. However, these responses were only observed after the second dose and lasted for 3 months, thus explaining the delayed peak of Covaxin action (33).

**Table 2:** List of studies on the purified inactivated SARS-CoV-2 vaccine Covaxin.

Study	Type of study	Number of participants	Study groups involved		Findings on immunogenicity/efficacy													Findings on safety	Additional comments
Mohandas et al. (23)	Non-human study	36 Syrian hamsters with 9 in each group	Group I -phosphate-buffered saline (PBS)		IgG negative till virus challenge IgG +ve in all by 14 DPI (OD=0.29) No NAb (PRNT_50_) response till 15 DPI Throat swab Viral gRNA copy number highest post challenge and persisted till 10 DPI													**Not mentioned in study**	3 Doses(Day 0,14,35) given. Vaccinated hamsters had lower weight loss following virus challenge. All vaccinated groups induced IgG2 and the NAb appeared at 3 weeks peaking at 7 weeks All vaccinated groups had normal morphology compared to congestive, fibrotic and haemorrhagic features in group I. No significant elevation of cytokines in vaccinated compared to IL-12 elevation in controls.
			Group II (BBV152C)- 6µg vaccine + Algel		IgG Ab in 3^rd^ week in 2 of 9 (OD=0.285) IgG Ab on day 48 in 9 of 9 (OD=0.55) Throat swab & trachea viral clearance on 7 DPI Lungs viral clearance on day 15 DPI														
			Group III (BBV152A)- 3µg vaccine + Algel-IMDG)		IgG Ab in 3^rd^ week in 8 of 9 (OD=0.42) IgG Ab on day 48 in 9 of 9 (OD=1.2) Highest observed NAb(PRNT_50_) (mean=28,810 at 7^th^ week) and post challenge mean=85,623) on 15 DPI Throat swab, lungs & trachea viral clearance on 7 DPI														
			Group IV (BBV152B)- 6µg vaccine + Algel-IMDG		IgG Ab in 3^rd^ week in 8 of 9 (OD=0.62) IgG Ab on day 48 in 9 of 9 (OD=1.32) Throat swab, lungs & trachea viral clearance on 7 DPI														
Yadav et al. (8)	Non-human primate study	20 Rhesus Macaques with 5 in each group	Covaxin 6μg+ Alum		IgG titer 1:1600-1:6400 NAb titer 1:87.4 - 1: 3974 Throat swab -viral clearance on 7 DPI BAL fluid viral clearance on 5 DPI													**No Adverse events noted**	2 dose (day 0,14) study. Necropsy Lung specimens negative for gRNA and sgRNA in vaccinated groups. Radiographic abnormalities resolved by 5 DPI in 2 vaccinated groups other than the 3µg + alum +imidazoquinoline group which showed No clinical or radiographic abnormalities. Resistance to pneumonia on Histopathological examination unlike placebo. Pro-inflammatory cytokines such as IL-6 were lower and anti-inflammatory cytokines such as IL-5 higher in all vaccinated.
			Covaxin 3μg + alum+ imidazoquinoline		Highest IgG titer (1:25600) Highest NAb titers of 1:209 to 1:5,217 Throat swab viral clearance on 7 DPI BAL fluid viral clearance on 5 DPI														
			Covaxin 6μg+ alum+ imidazoquinoline		IgG titer 1:1600-1:6400 NAb titer 1: 29.5 -1: 3403 Throat swab viral clearance on 7 DPI BAL fluid viral clearance on 5 DPI														
			Placebo		No NAb and IgG response Throat swab viral clearance not seen even by 7 DPI BAL fluid viral clearance not seen even by 7 DPI														Chest X-ray showed infiltrates, bronchopneumonia, or lobar pneumonia which persisted till 7 DPI.
Ganneru et al. (7)	Pre-clinical study on 3 animal species		BALB/c mice at 1/20^th^ or 1/10^th^ Human single dose + adjuvant (intra-peritoneally)		High Ag Binding and NAb titers(PRNT_90_) (100% seroconversion) Day 7 -10^2^ titer Day 14 – 10^3^ titer Day 21 – 10^4^ titer Day 28 – peak titer Adjuvanted (algel/algel-IMDG) formulations elicited high Ab levels targeted against S1 compared to non-adjuvanted and lasted 98 days.													Only local reactogenicity observed which was self resolving	Algel-IMDG found non-mutagenic and well tolerated at test as well as repeat dose in the 3 animal models. The TLR7/8 agonist adjuvant supports Th1-biased Ab responses and has high IgG2a/IgG1 ratio, IFN-γ response compared to algel.
			New Zealand White rabbits		High NAb titers (PRNT_90_ as well as MNT_50)_ comparable to convalescent human sera Day 21- 10^4^ titer (100% seroconversion)														
			Wistar rats & Swiss Albino mice															Maximum Tolerated dose (=Human single dose) and repeated dose elicited no ill-effects	
Zare et al. (10)	Cross-sectional study on health workers	503 Health care workers given atleast 1 of 3 different vaccine	Covaxin (42)		None performed													92.9 % had side effects. Injection site pain (83.7%) >Fatigue (41.9%) >headache (27.9%)	Injection site pain was most common in all three vaccines. AZD1222 had highest %age of systemic side-effects. Prevalence rate of complications in Covaxin not significantly different from Sputnik-V and AZD1222. No serious/life-threatening side effects observed in all 3 vaccine groups. Side-effects disappeared by 7 days post-innoculation in all groups.
			Sputnik-V(238)															81.9 % had side-effects. Injection site pain (56.7%) >Muscle pain (41.6%) >Fever & chills (37.4%)
			AZD1222 (223)															88.8 % had side-effects. Injection site pain (70%) >Fatigue (68.4%) >fever & chills (67.1%)
Singh et al. (COVAT study) (26)	Cross-sectional study of Health care workers	96 (1^st^ dose) 90 (2^nd^ dose)	Covaxin		Seropositivity after 1^st^ dose- 43.8% GMT’s after 1^st^ dose- 16.8 AU/mL. Seropositivity after 2^nd^ dose- 80% GMT’s after 2^nd^ dose- 48.3 AU/mL.													31.2% mild-moderate side-effects after 1^st^ dose and 11.1% after 2^nd^ dose. Breakthrough infections in 2.2%. No serious AEFI	People with comorbidity especially Type 2 Diabetes had lower seropositivity in both vaccines. Past history of infection resulted in overall significantly higher seropositivity vis-à-vis unexposed individuals. Females also had 9% higher seropositivity. Covishield had significantly increased seropositivity, NAb titer after 1^st^ dose while Covaxin required 2 doses to achieve significant effect.
		456 (1^st^ dose) 425 (2^nd^ dose)	Covishield		Seropositivity after 1^st^ dose- 86.8% GMT’s after 1^st^ dose- 62.4 AU/mL. Seropositivity after 2^nd^ dose- 98.1% GMT’s after 2^nd^ dose- 129.3 AU/mL.													Higher mild-moderate side-effects 46.7% after 1^st^ dose and 18.1% after 2^nd^ dose. Breakthrough infections in 5.5%. No serious AEFI.
Singh et al. (COVAT study follow-up) (30)	6-month longitudinal study	74	Covaxin		Anti- spike GMT (AU/ml) (SARS-CoV-2 naïve cohorts) 21 days post 1^st^ dose- 16.17 21 days post 2^nd^ dose- 50.11 3 months post 2^nd^ dose- 50.81 6 months post 2^nd^ dose- 46.27 Anti- spike GMT declined by only 8% at 6 months compared to peak titer. Seropositivity (%) (SARS-CoV-2 naïve cohorts) 21 days post 2^nd^ dose- 77 3 months post 2^nd^ dose- 55.7 6 months post 2^nd^ dose- 37.7													Not mentioned in study	Covaxin showed lower seropositivity and anti-Spike GMT compared to Covishield at all time points but with much less decline from peak titers at 6 months after 2^nd^ dose. Breakthrough infection rates were similar in the 2 vaccines Covishield (54/407, 13.3%) vs Covaxin (10/74, 13.5%).
		407	Covishield		GMT (AU/ml) (SARS-CoV-2 naïve cohorts) 21 days post 1^st^ dose- 61.93 21 days post 2^nd^ dose- 132.88 3 months post 2^nd^ dose- 112.78 6 months post 2^nd^ dose- 73.83 GMT declined by 44% at 6 months compared to peak titer. Seropositivity (%) (SARS-CoV-2 naïve cohorts) 21 days post 2^nd^ dose-98.7 3 months post 2^nd^ dose- 92.6 6 months post 2^nd^ dose- 22.1														
Sharma et al. (29)	Cross-sectional study of Health care workers	168(Atleast 1 dose) 154 (Both doses)	Covaxin		None performed													33 infections of 168 (19.6%) Breakthrough 24 of 154 (15.6%)	History of prior infection with COVID-19 and atleast one vaccine dose was significantly protective of breakthrough infections.
		157(Atleast one dose) 125 (Both doses)	Covishield															24 infections of 157 (15.3%) Breakthrough 13 of 125 (10.4%)	
Dash et al. (32)	Cross-sectional study including breakthrough cases	35	Covaxin		Anti-Spike receptor binding domain IgG Ab - 27 (77.1%) Ab titer- 213.5 AU/ml [interquartile range (IQR)537.5]													Symptomatic-29 (82.9%) Asymptomatic- 6 (17.1%) Hospitalized -3 (8.6%)	Seropositivity in Covishield vaccinees was significantly higher than Covaxin. Among the 27 (breakthrough infection) hospitalised vaccinees, 1 (Covishield recipient) died.
		239	Covishield		Anti-Spike receptor binding domain IgG Ab - 231 (96.7%) Ab titer- 647.5 AU/ml (IQR: 1645.1),													Symptomatic-199 (83.3%) Asymptomatic – 40 (16.7%) Hospitalized – 24 (10%)
Kumar et al. (35)	Prospective cohort study of health care workers	44	Covaxin		Increased induction of Type 1,2,17 and pro-inflammatory cytokines (IFN-γ, IL-1a,IL-1b, IL-2, IL-3, TNF-α, IL-4, IL-5,IL-6, IL-7 IL-10, IL-12, IL-13,IL-17A). Reduced synthesis of IL-25, IL-33, GM-CSF, Type 1 interferons. Increased plasma levels of chemokines (CCL4, CXCL1, CXCL2 and CX3CL1). Reduced levels of CXCL10. Significant correlations between IL-2, IL-17A, IL-4, IL-5 and NAb at baseline.													**Not mentioned in study**	The effect of ‘Prime boost’ Covaxin on cytokine and chemokine profiles was studied at baseline(0) and after 1,2 and 3 months. Raised type 1,17 and pro-inflammatory cytokine levels show ‘immune memory induction’ while raised type 2 cytokines maybe attributed to vaccine adjuvant. Raised chemokines also show innate immunity induction.
Yadav et al. (39)	Cross-sectional study	17	Covaxin vaccinees(28 days after 2^nd^ dose)		Geometric mean titer (GMT) of serum against B.1- 187.5 (95% CI: 129.3–271.9), Beta- 61.57 (95%CI: 36.34–104.3) Delta - 68.97 (95%CI: 24.72–192.4)													**Not mentioned in study**	Neutralization of sera by covaxin recipients was assessed and compared with sera of recovered patients against Beta and Delta variants.
		20	COVID recovered(5-20 weeks after infection)		GMT of sera against B.1 - 97.8 (95%CI: 61.2–156.2) Beta- 29.6 (95%CI: 13.4–65.0) Delta- 21.2 (95% CI: 6.4–70.1)														
Sapkal et al. (36)	Cross-sectional study	42	Covaxin		GMT of IgG: For S1-Receptor Binding domain protein- 2250 For N Protein- 3099 GMT-Nab by PRNT_50_ (for prototype D614G)- 337.5 GMT by PRNT_50_ (For the B.1.1.28.2 variant)- 175.7													**Not mentioned in study**	IgG levels and NAb activity were assessed and it was concluded that a 2 -dose BBV152 is effective against both B.1.1.28.2 variant and D614G prototype( which was used to develop Covaxin), compared to protection afforded by natural infection.
		Total (n=19) B.1.1.7 (n=2) B.1.351 (n=2) B.1.1.28.2 (n=2) B1 lineage (n=13)	Convalescent sera (15–113 days after positive report).		GMT of IgG: For S1-Receptor Binding domain protein- 794.8 For N Protein- 4627 GMT NAb by PRNT_50_ (for prototype D614G)- 120.1 GMT by PRNT_50_ (For the B.1.1.28.2 variant)- 109.2														
Sapkal et al. (37)	Cross-sectional study		38 vaccine recipients		Nab titers by PRNT_50_ of vaccinee sera had comparable efficacy against UK variant of GR clade (mutant hCoV-19/India/20203522) as well as the hCoV-19/India/2020770 (used for developing Covaxin) belonging to G clade and hCoV- 19/India/2020Q111 belonging to O clade. Median ratio of 50% neutralization- 0.8 hCoV- 19/India/2020770 vs UK variant Median ratio of 50% neutralization- 0.9 hCoV- 19/India/2020770 vs hCoV- 19/India/2020Q111													**Not mentioned in study**	PRNT_50_ values from the different groups did not show any significant difference (P>0.05).
Yadav et al. (40)	Cross-sectional study involving various categories of Covaxin recipients				GMT of NAb (PRNT_50_)													**Not mentioned in study**	Neutralization was assessed against Delta, Delta AY.1 and B.1.617.3 compared with B.1 variant. NAb titers for BTI group was highest followed by CRV and CNV. In the CNV group, compared to B1, NAb titer against B.1.617.3 was lowest at a 1.88 reduction while Delta showed 1,29 reduction. The CRV and BTI groups also showed a similar pattern of reduction of B.1.617.3>Delta AY.1>Delta, although there were higher fold reductions in neutralization. The role of memory cells could explain the high titers observed in CRV and BTI groups compared to CNV group. Although titers against the new variants were reduced, some protection against severe disease could still be plausible.
		42	COVID-19 naïve vaccinees **(CNV)**		Delta			Delta AY.1					B.1.617.3		B1				
					241.6 (95% CI: 167.8–347.7)			209.1 (95% CI: 146.5–298.3)					165.3 (95% CI: 115.6–236.5)		310.6 (95% CI: 222–434.6)				
		14	COVID-19 recovered and vaccinated **(CRV)**		Delta			Delta AY.1					B.1.617.3		B1				
					328.6 (95% CI: 186.9–577.9)			234.5 (95% CI: 138.7–396.4)					217.8 (95% CI: 136.7–347.1)		820.1 (95% CI: 469–1434)				
		30	Breakthrough infections after vaccination **(BTI)**		Delta			Delta AY.1					B.1.617.3		B1				
					465.6 (95% CI: 213.2–1016)			317.2 (95% CI: 125.5–801.4)					259.7 (95% CI: 157.1– 429.4)		896.6 (95% CI: 550.3–1461)				
Kumar et al. (41)	Cross-sectional study involving health care workers	84									SARS-COV-2 IgG proteins (AU/ml)				NAb % inhibition-			**Not mentioned in study**	A single dose of Covaxin administered to previously SARS-COV-2 infected individuals could elicit comparable humoral immune response to that seen in non-exposed individuals administered both doses of the vaccine.
			Vaccinees with no prior infection		Baseline						IgG N- 0.71 IgG S- 0.37				-1.43				
					Month 1						IgG N- 2.4 IgG S- 2.3				9.2				
					Month 2						IgG N- 56.3 IgG S- 86.7				68.9				
		30	Vaccinees with prior infection		Baseline						IgG N- 29.3 IgG S- 48.8				74.1				
					Month 1						IgG N- 78.6 IgG S- 167.2				95.8				
					Month 2						IgG N- 95 IgG S- 211				94.5				
Kant et al. (43)	Cross-sectional study involving 98 vaccine recipients	18	Vaccinees given Covishield + Covaxin		GMT S1-RBD ELISA titer		GMT N protein ELISA Titer						IgG (GMT) inactivated SARS-CoV-2 virus		NAb (PRNT_50_) GMT, Against B1/ Alpha/ Beta/ Delta			**Inj. Site Pain** - 11.1% after 1^st^ dose none after 2^nd^ dose **Pyrexia**- 27.7% after 1^st^ dose and 11.1% after 2^nd^ dose **Malaise**- 33.3% after 1^st^ dose and 5.5% after 2^nd^ dose	Pain at injection site was the most common local adverse effect while most common systemic adverse events were pyrexia and malaise. All adverse events in the Covaxin + Covishield group were comparable to either group alone. Covishield vaccinees were found to have the highest titers for GMT S1-RBD. The heterologous group had highest GMT N protein and IgG (GMT) to inactivated virus titer as well as highest levels of NAb (GMT) towards all the 4 variants.
					1866		1145					171.4			**B1**- 539.4 **Alpha**- 396.1 **Beta**- 151 **Delta**- 241.2				
		40	Covishield vaccinees		2260		353.7						111		**B1**- 162 **Alpha**- 122.7 **Beta**- 48.43 **Delta**- 51.99			**Inj. Site pain-** 5% after 1^st^ dose 5% after 2^nd^ dose **Pyrexia**- 20% after 1^st^ dose and 15% after 2^nd^ dose **Malaise**- 5% after 1^st^ dose and 5% after 2^nd^ dose	
		40	Covaxin vaccinees		710		742.4						86		**B1**- 156.6 **Alpha**- 112.4 **Beta**- 52.09 **Delta**- 54.37			**Inj. Site pain -** 7.5% after 1^st^ dose 7.5% after 2^nd^ **Pyrexia**- 30% after 1^st^ dose and 15% after 2^nd^ dose **Malaise**- 32.5% after 1^st^ dose and 15% after 2^nd^ dose	
Basavaraja et al. (27)	Prospective observational study	9292 doses to 5986 vaccinees	Covishield		**Not mentioned in study**													Incidence rate of adverse events was 4.32%. 433 AE (409 expected as per factsheets) 94.22%- associated with immunization of which 78.98% related to vaccine products and 15.24% due to anxiety.	Half (50.9%) vaccinees had a single AE, 34.9% had 2 AE’s while 8.6% reported 3 AE’s. Most of the AE’s followed the 1^st^ dose of vaccination. Covishield vaccinees had mostly fever, injection site tenderness, pain and joint pain muscle aches, while Covaxin recipients had injection site pain, fever and 3 cases had giddiness which was not mentioned in factsheets.
		2364 doses to 1749 vaccinees	Covaxin															Incidence rate of adverse events was 0.57%. 12 AE (9 expected as per factsheets) 8(66.6%)- associated with immunization of which none related to vaccine products and all related to anxiety.	
Choudhary et al. (28)	Longitudinal Cohort study involving Health Care workers	308	Covishield		Highest levels of spike >protein IgG observed in the 12^th^ week (median=1299.5 AU/ml) (IQ:517.9-5019.2) falling to 637.2 AU/ml (IQ: 186.5–3,055.3) after 6 months. In unexposed seronegative individuals, 81.9% had seroconversion at 4 weeks after 1^st^ dose.													Out of 81 breakthrough infections, 37% were Covishield recipients.	Covishield vaccinees had significantly higher IgG Ab compared to Covaxin . There was a 2-fold reduction in spike Ab titers in Covishield while Covaxin vaccinees had a more drastic 4-fold reduction.
		306	Covaxin		Highest levels of spike protein IgG observed in the 12^th^ week (Median= 342.7 AU/ml) (IQ: 76.1–892.8) falling to 95.1 AU/ml (IQ: 36.5–277.2) at 6 months. In unexposed seronegative individuals, 16.1% had seroconversion at 4 weeks after 1^st^ dose.													Out of 81 breakthrough infections, 63% were after Covaxin.
Desai et al. (38)	Test negative case-control study	1068 matched case-control pairs			Adjusted effectiveness of 2 doses of Covaxin against symptomatic RTPCR positive (tested atleast 2 weeks after 2^nd^ dose) SARS-CoV-2 was 50% (95% CI 33-62) and if testing was at 4 weeks or more, the adjusted effectiveness was 46% (95% CI 22–62). At 6 weeks effectiveness rose to 57% (95% CI 21–76). If participants with prior infection were excluded the adjusted effectiveness was 47% (95% CI 29–61).													**Not mentioned in study**	This study was undertaken at a time of surge in cases during the second wave of COVID in India. The Delta variant was infamous for its immune evasion and might have been responsible for the lower efficacy compared to phase III trials conducted by Bharat Biotech.
Medigeshi et al. (42)	Cross-sectional study	Median duration from 2nd dose of either vaccine- 234 days			GMT of Focus reduction neutralization titer (FRNT_50_)							Neutralisation titers above limit of quantification (1:20)Against Omicron						**Not mentioned in study**	Both Covaxin and Covishield vaccinees with no prior infection had a ~26-fold reduction in FRNT_50_ titers against Omicron compared to ancestral variant after 6 months. Those with prior infection had ~57-fold reduction. Significant reduction in neutralizing ability of both vaccines was observed but prior infection was associated with significantly high titers. Anti-nucleocapsid Ab wane in Covaxin vaccinees, however, those with prior infection sustain Ab for longer periods compared to Covishield.
					Ancesrtral	Delta					Omicron								
		20		Only Covaxin	380.4	164.7					14.3	5 out of 20 samples							
		20		Covaxin + previous infection	806.1	260.2					14.12	6 out of 20 samples							
		20		Only Covishield	379.3	11.9					14.7	5 out o 20 samples							
		20		Covishield + previous infection	1526.2	358.1					26.3	9 out of 20 samples							
Houshmand et al. (31)	Cross-sectional study																	**Side-effect intensity/incidence**	
		578		Covishield														98.6 % had atleast one side effect. Highest intensity of almost all side effects	No serious side-effects were reported for BBV152. Adenovirus-vector based vaccines were found to cause higher levels of side-effects attributable to cytokine/chemokine release compared to inactivated vaccines. 73.1% side-effects observed within 24 hours for all vaccines.
		25		Covaxin														100% had atleast 1 side-effect. Local pain in the hand only side-effect of significant intensity.	
		426		GAM-Covid-Vac														93.2% had atleast 1 side-effect. Injection site pain, Fever, muscle pain common.	
		102		BBIP-CorV														87.3% had at least one side effect. Lowest intensity of almost all side effects	
Sapkal et al. (44)		17		CS/CV	S1-RBD IgG Ab titer- 4.13 fold reduction in GMT mean titer ratio of 1^st^ and 6^th^ month Reduction in Ratio of GMT of NAb at 1^st^ and 6^th^ month													Not mentioned	Heterologous vaccinees had higher NAb titers despite significant fold reductions in titers 6 months after 2^nd^ dose. Comparison with B.1 ancestral variant revealed that NAb titers were drastically low for omicron variant.
					B.1 (ancestral)					Alpha			Beta				Delta		
					7.17					6.98			7.19				5.75		
					Reduction in NAb titers in comparison with B.1 for different VOCs														
					Alpha					Beta			Delta				Omicron		
					1.28					3.43			1.75				19.16		
		36		Covishield	S1-RBD IgG Ab titer- 6.8 fold reduction in GMT mean titer ratio of 1^st^ and 6^th^ month Reduction in Ratio of GMT of Nab at 1^st^ and 6^th^ month														
					B.1 (ancestral)					Alpha			Beta				Delta		
					2.87					3.51			2.76				1.96		
					Reduction in NAb titers in comparison with B.1 for different VOCs														
					Alpha					Beta			Delta				Omicron		
					1.63					3.43			2.27				23.15		
		35		Covaxin	S1-RBD IgG Ab titer- 4.87 fold reduction in GMT mean titer ratio of 1^st^ and 6^th^ month Reduction in Ratio of GMT of Nab at 1^st^ and 6^th^ month														
					B.1 (ancestral)					Alpha			Beta				Delta		
					3.17					3.72			2.61				3.36		
					Reduction in NAb titers in comparison with B.1 for different VOCs														
					Alpha					Beta			Delta				Omicron		
					1.67					2.56			2.83				24.21		
Malhotra et al. (33)	Retrospective cohort study involving previously infected HCWs				Estimated vaccine effectiveness against:														Full vaccination with BBV152 was associated with a good protective effect while partial vaccination was ineffective. Since most of the reinfections occurred during the Delta variant-induced 2^nd^ wave, Covaxin accorded sufficient protection in pre-infected participants.
					Reinfection						Symptomatic reinfection			Asymptomatic reinfection					
		1089		Fully vaccinated with Covaxin	86%						87%			84%					
		356		Partially vaccinated with Covaxin	12%						16%			–					
		472		Unvaccinated	–						–			–					

### Response of Covaxin against SARS-CoV-2 variants

Sufficient action of vaccines against newer variants is essential to reduce mortality and control the spread of infection to manageable levels. The ameliorative action of Covaxin against several variants has been tested. A study by Sapkal et al. reported higher GMT of IgG for S1-receptor binding domain protein as well as higher NAb GMT for the B.1.1.28.2 and D614G strains in Covaxin vaccinee sera compared to convalescent sera (35). In another study by Sapkal et al., NAb titers of vaccinee sera had comparable efficacies against GR, G, and O clades of SARS-CoV-2 and could effectively neutralize the Alpha variant (36). Although BBV152 elicited comparatively reduced titers against the Delta and other newer variants, some rudimentary protection was still afforded. Desai et al. undertook their study at the peak of the second wave in India, probably triggered by the evasive Delta variant. They found an adjusted effectiveness against symptomatic infection to be 50% at 2 weeks after the second dose of BBV152, which rose to a somewhat reasonable figure of 57% at 6 weeks. A strong Th1 bias also allayed fears of serious adverse events (40). Ella et al. had reported efficacy of 65.2% against the Delta variant in the phase 3 trials (25). The study by Malhotra et al. conducted during the wave triggered by the Delta variant reported significant effectiveness (86%) of a two-dose Covaxin regimen (43) Yadav et al. observed neutralization of sera by Covaxin recipients in comparison with those of recovered patients and observed significantly higher levels of GMT against ancestral (B.1), Beta and Delta variants in vaccinees in comparison to the unvaccinated suggesting a somewhat ample coverage of these variants by the vaccine (34). In another study by the same author, action of Covaxin against B.1, Delta, Delta AY.1, and 1.617.3 was assessed, and it was inferred that a milder level of protection was nonetheless afforded by the vaccine against the newer variants (37). Higher titers were also observed in vaccinees who had been previously infected compared to vaccinees without any prior exposure. In fact, a significant humoral response was also observed by Kumar et al., who observed that a single dose of BBV152 administered to previously infected individuals had comparable effectiveness to non-exposed vaccinees administered both doses. IgG Ab against Spike proteins in individuals administered a single dose of Covaxin were markedly elevated (28 days after the first dose) at 167.2 AU/ml in recipients with prior viral exposure in comparison to just 2.3 AU/ml in those with no prior infections. However, the difference in titers between the two groups was less significant after two doses. Kumar et al. thus advocated saving on valuable vaccine doses by giving only a single dose of Covaxin to previously infected individuals; instead reserving the two-dose regimen for non-exposed individuals (38).

### Response of Covaxin against the B.1.1.529 (Omicron) variant

Covaxin acted poorly against the B.1.1.529 variant and consequently, immune escape appeared widespread. Covishield fared no better. Despite extensive coverage of vaccination campaigns in the Indian subcontinent utilizing both the above vaccines, there were widespread incidences of breakthrough infections and reinfections **(** **Table 2** **)**. Recipients of both vaccines with no prior virus exposure had a ~26-fold reduction in neutralization titers (FRNT_50_) against Omicron compared to the ancestral variant, 6 months after the second dose. However, those who had a history of prior exposure to infection had significantly higher titers, albeit these subsided twice as rapidly. Interestingly, Covaxin recipients sustained anti-nucleocapsid antibodies for longer periods as compared to Covishield (41).

### Heterologous prime boost vaccination

The study by Kant et al. observing the serendipitous ‘mix and match’ of Covaxin and Covishield reported the lowest Geometric mean titers (GMT) for both the S1-receptor binding domain antibodies as well as antibodies to the inactivated virus with Covaxin. However, neutralizing antibodies against B1, Alpha, Beta, and Delta were comparable to that observed with Covishield. Interestingly, mixing the two vaccines yielded better results than either vaccine taken alone. The heterologous group reported the highest titers for the N (nucleocapsid) protein and IgG to the inactivated virus. NAb’s against the four variants were also significantly higher than that seen in homologous groups. Nonetheless, neutralization of the sera of BBV152 vaccinees measured in geometric mean titer against the B1, Beta, and Delta variants was significantly higher than that seen with sera from recovered patients (39). Sapkal et al. assessed sera of vaccinees who had received heterologous vaccination (first dose Covishield, second dose Covaxin) and despite significant-fold reductions in GMT of NAb 6 months after the second dose, the heterologous group had consistently higher titers compared to the groups receiving homologous vaccination (either Covishield or Covaxin). NAb titers against the Omicron variant were remarkably reduced for both heterologous/homologous vaccination compared to ancestral, Alpha, Beta, and Delta variants. Nonetheless, heterologous vaccination was immunogenically superior to the homologous mode of vaccination (42).

Heterologous prime-boost vaccination was similarly encouraged by other studies which claimed higher inductions of immunogenicity with combinations of vector-based + inactivated vaccines, which suggests great scope for such regimens in tackling newer variants (45).

## Conclusion

After a perusal of the studies included in the systematic review, the authors found the safety profile of Covaxin to be satisfactory and comparable with data from other vaccines, most of the complaints being of injection site pain. A study reported milder adverse effect profile of inactivated vaccines such as Covaxin compared to viral-vector-based ones. Although some studies reported slightly more breakthrough infections with the vaccine compared to other candidates, none of the studies reported any serious/severe adverse events or fatalities. Immunogenicity performance of BBV152, albeit higher than the natural immunity of recovered patients, with the added advantage of being Th1-cell biased, was not as competitive as Oxford–AstraZeneca’s AZD1222 (Covishield), as the latter consistently showed higher seroconversion rates and NAb titers. Covaxin displayed lower immunogenic parameters at almost all time points after the second dose, with titers usually lagging behind those seen with Covishield. While AZD1222 showed significant immunogenicity after the first dose itself, it required generally two doses of Covaxin to impart sufficient immunity. Previously infected individuals nonetheless showed good results with the administration of a single dose of Covaxin. Individuals with prior viral exposure administered at least two doses of Covaxin had the best results. In all, binding and neutralizing antibody titer values for Covaxin were not very impressive. Although some protection was afforded against strains such as Alpha, Beta, and Delta, it was not substantial. Neither Covaxin nor Covishield could provide sufficient immunity against the Omicron strain. However, a vaccination regimen including both vaccines displayed better immunogenicity, especially against multiple strains. Further experimentation with heterologous boost vaccination may be beneficial in tackling future variants.

## Data availability statement

The original contributions presented in the study are included in the article/supplementary material. Further inquiries can be directed to the corresponding author.

## Author contributions

Conceptualization, TIA and SM; methodology, validation, TIA and SM; formal analysis, investigation, data curation, writing—original draft preparation, TIA and SM; writing—review and editing, supervision, TIA, SR, SI, JA, KH and SM. All authors have read and agreed to the published version of the manuscript.

## Conflict of interest

The authors declare that the research was conducted in the absence of any commercial or financial relationships that could be construed as a potential conflict of interest.

## Publisher’s note

All claims expressed in this article are solely those of the authors and do not necessarily represent those of their affiliated organizations, or those of the publisher, the editors and the reviewers. Any product that may be evaluated in this article, or claim that may be made by its manufacturer, is not guaranteed or endorsed by the publisher.
